# MTAP-Null Tumors: A Comprehensive Review on Synthetic Vulnerabilities and Therapeutic Strategies

**DOI:** 10.3390/cells14241964

**Published:** 2025-12-10

**Authors:** Bavani Subramaniam, Wai Chin Chong, Aylar Babaei, Miriam Bornhorst, Chunchao Zhang, Roger Packer, Javad Nazarian

**Affiliations:** 1 Brain Tumor Institute, Center for Cancer and Immunology Research, Children’s National Hospital, Washington, DC 20012, USA; bsubramani@childrensnational.org (B.S.);; 2 Northwestern University Feinberg School of Medicine, Ann & Robert H. Lurie Children’s Hospital of Chicago, Chicago, IL 60611, USA; 3 Children’s Research Center, University Children’s Hospital Zurich, 8008 Zurich, Switzerland

**Keywords:** methylthioadenosine phosphorylase, CDKN2A, cancer, metabolism, therapy

## Abstract

Homozygous deletion of the 9p21.3 genomic locus spanning the *CDKN2A/B* and *MTAP* genes is an event affecting 15% of cancers. While *CDKN2A* is a well-established tumor suppressor gene, the role of *MTAP* in tumorigenesis varies across cancer types. *MTAP* codes for methylthioadenosine phosphorylase, a key enzyme in the methionine salvage pathway, and its loss has been associated with several downstream synthetic vulnerabilities. Despite multiple efforts to exploit MTAP loss for targeted therapies, none of these efforts have yielded substantial results in clinical trials. In this review, we consolidate the existing literature along with our systematic analysis to provide an updated perspective on the incidence of MTAP loss in different cancers and elucidate its impact on metabolism, immune microenvironment, and tumor progression. In addition, we summarize the therapeutic strategies that have been investigated preclinically on MTAP-null tumors before and after the advent of functional genomic screening tools. We further assess the current landscape of clinical trials investigating MTAP-targeted inhibitors, evaluating their limitations and potential avenues for improvement. The insights gained from this review will inform future research directions beyond the promising PRMT5/MAT2A axis for rational combination therapies that would work synergistically to eradicate this devastating disease.

## 1. Introduction

The discovery of the methylthioadenosine phosphorylase (MTAP) enzyme in malignant murine hematopoietic cells in 1977 was a significant scientific milestone that laid the groundwork for future targeted therapies [1]. MTAP was found to be absent in certain cancer cells that also showed increased dependency on methylthio groups for cell division, thus establishing its role as an important metabolic enzyme [2]. Over the years, several other human cancers including leukemias and solid tumors showed absence of MTAP, prompting researchers to dive deeper into comprehending its role in oncogenesis [3,4]. In 1984, using mouse-human somatic cell hybridization, the location of the *MTAP* gene was assigned to human chromosome 9, specifically between the regions 9p and 9q [5]. A decade later, the cyclin-dependent kinase 2A (*CDKN2A*) gene was identified at the 9p21 region, which is homozygously deleted in multiple cancers [6]. In recent years, the Pan-Cancer Analysis of Whole Genomes (PCAWG Consort 2020) mapped the 9p21.3 genomic locus as having the most common biallelic somatic copy number alteration, affecting an estimated 15% of cancers [7]. These findings prompted several efforts to study the genes encompassed within this region for their tumor suppressor roles or for targeted therapy. While *CDKN2A/B* genes have been established as tumor suppressor genes [8,9], the role of *MTAP* in tumorigenesis has seen contradicting perspectives [10,11]. However, over the years, it has become widely accepted that *MTAP* deletion offers a significant potential for targeted therapy in cancers with 9p21.3 loss [7,12,13]. The versatility of MTAP in exposing vulnerabilities in cancer cells warrants a fresh perspective to reevaluate multiple therapies investigated over the years. In this review, we will explore the incidences of MTAP deficiency in different malignancies, elucidate its biological consequences, and summarize the synthetic vulnerabilities associated with it in order to expedite breakthroughs in targeted therapy.

## 2. Loss of MTAP and Disease Overview

### 2.1. A Versatile Biomarker for Targeted Therapy

**Figure 1:**
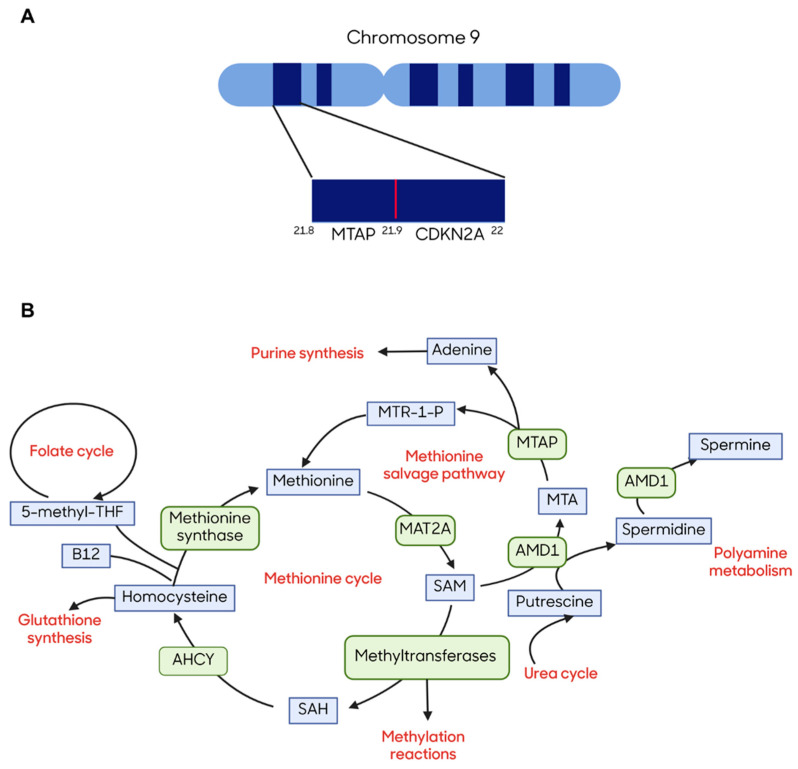
Methylthioadenosine phosphorylase and its mechanism of action. (**A**) Chromosome location of *MTAP* and *CDKN2A* on the 9p21.3 locus indicates their proximity towards each other. Created in BioRender. Bavani Subramaniam. (2025) https://app.biorender.com/illustrations/67b3b8ed7aef64c47ac7a102. (**B**) MTAP is an enzyme in the methionine salvage pathway, which plays a crucial role in catalyzing the conversion of MTA to MTR and adenine. Created in BioRender. Bavani Subramaniam. (2025) https://app.biorender.com/illustrations/67b3bb9f9aa19f5100ee2a5a. Abbreviations: AHCY, adenosylhomocysteine; AMD1, adenosylmethionine decarboxylase 1; MAT2A, methionine adenosyltransferase 2A; MTA, methylthioadenosine; MTAP, methylthioadenosine phosphorylase; MTR-1-P, 5-methylthioribose-1-phosphate; SAH, S-adenosyl-homocysteine; SAM, S-adenosylmethionine; THF, tetrahydrofolate.

MTAP plays a significant role in the methionine salvage pathway to catalyze the conversion of methylthioadenosine (MTA) into 5-methylthioribose-1-phosphate (MTR-1-P) and adenine, which are further converted into methionine and adenosine monophosphate (AMP), respectively [7,13]. Loss of function mutation in the *MTAP* gene is caused by homozygous deletion of the 9p21 chromosomal locus that also encompasses the tumor suppressor genes *CDKN2A/B* (Figure 1A) [14,15,16]. Considering that the incidences of *MTAP* and *CDKN2A/B* co-deletion are events affecting 15% of cancers [17], *MTAP* has a high potential of being exploited for targeted therapy. Loss of the MTAP enzyme leads to disruption in the methionine salvage pathway that initiates a cascade of downstream metabolic vulnerabilities (Figure 1B) [7,18]. In addition, current therapeutics targeting *CDKN2A* loss using cyclin-dependent kinase 4/6 (CDK4/6) inhibitors are fraught with resistance mechanisms involving downstream cell cycle kinases [19]. Furthermore, it has been established that 9p21 deletions are early and clonal events in different cancers, reducing the risk of tumor heterogeneity and improving efficacy of targeted therapies for these tumors [17,20].

### 2.2. Methods for Detection of MTAP Loss

Detection of MTAP loss relies on identifying tumors with homozygous deletion of *MTAP*. This is achieved through immunohistochemical (IHC) assays, next-generation sequencing (NGS), and fluorescence in situ hybridization (FISH) [21,22]). IHC for MTAP deletion can help indicate the presence of homozygous *MTAP* deletion and be used as a surrogate for CKDN2A loss (78% sensitivity, 96% specificity) [23]. However, tissues used for IHC are susceptible to damage and impaired fixation, which can affect detection accuracy [24]. Additionally, MTAP false positive detection can occur in tissues with few tumor cells due to intratumoral lymphohistiocytic infiltrates [23]. These issues can be mitigated by selecting sections that accurately represent tumor cells and correlating IHC slides with matched hematoxylin-and-eosin-stained slides [25].

NGS has been incorporated in clinical pathology labs and clinical trials targeted for *MTAP*-deleted tumors including the recent trial on AMG193 (NCT05094336) [26,27]. Despite its utility, copy number variation (CNV) analysis using panel-based sequencing faces challenges in accurately determining the exact copy number due to the proportion of tumor cells in the tissue and the lack of standardized thresholds for amplification and deletion [28,29,30]. Additionally, relying on *CDKN2A* copy number loss is not always feasible as not all tumors with *CDKN2A* loss exhibit homozygous *MTAP* deletion. Hence, CNV analysis has to incorporate *MTAP* as a gene of interest and may be accompanied by IHC validation to ensure accurate detection of MTAP loss.

FISH of *CDKN2A* homozygous deletion has also been incorporated for diagnosis [21,22]. However, FISH has limitations in routine application due to a lack of technical knowledge, resource constraints, and time-consuming procedures [25]. Concurrent detection of *CDKN2A* and *MTAP* is particularly challenging due to differences in gene size and the potential for missed *CDKN2A* microdeletions in intact-*MTAP* cases [31,32]. Higher-resolution detection of *CDKN2A* and *MTAP* deletion requires smaller FISH probes, which may have lower hybridization efficiency [32]. To mitigate this shortfall, employment of MTAP IHC as a surrogate for *CDKN2A* FISH has shown increased sensitivity and accuracy in diagnosis [23].

In a number of other cancers, such as lymphomas, gastric cancers, hepatocellular carcinoma (HCC), and nasopharyngeal carcinomas, reduced MTAP expression is associated with promoter hypermethylation, which produces similar functional loss as tumors with homozygous deletion of the *MTAP* gene [33,34,35,36,37]. *MTAP* promoter hypermethylation can be detected using methylation-specific polymerase chain reaction (PCR) [34], though clinical reports on this technique for MTAP detection are limited. Besides that, multiplex ligation-dependent probe amplification (MLPA) to detect copy number alterations (CNAs) from cell-free DNA extracted from cerebrospinal fluid (CSF) has been used to diagnose homozygous *CDKN2A* loss [38] and could potentially be improvised to detect *MTAP* deletion in future studies. Researchers are also advocating for the use of droplet digital PCR (ddPCR) for detecting *CDKN2A* and *MTAP* homozygous deletion in malignant pleural mesothelioma (MPM), citing its advantages of being less expensive, less time-consuming, and technically easier than FISH [39].

### 2.3. Incidence of MTAP Loss

Loss of MTAP has been reported by multiple studies spanning different cancer types, including through the utilization of the PCAWG Consort [7]. These studies have established that homozygous deletion of *MTAP* in glioblastoma (GBM) is the most common occurrence, affecting about 30–50% of cases [7,40]. This is followed by bladder cancer at about 35–40% and pancreatic cancers at 30% [7,41,42]. *MTAP* loss has also been reported in 17.1% of gastrointestinal (GI) tumors and 15% of lung cancers [43,44]. Other cancers such as bile duct, skin, soft tissue, and head cancer, as well as osteosarcoma, make up about 10–25% of cases [7]. Bone cancers, breast cancers, HCC, and colorectal cancers are some others that have reported less than a 10% frequency of homozygous *MTAP* deletion [7,45,46].

To build on these findings, we conducted an integrative analysis of complete homozygous deletion and low expression of *MTAP* using data from two major cancer genomics resources, the Cancer Cell Line Encyclopedia (CCLE DepMap Public 24Q4) and The Cancer Genome Atlas (TCGA) through cBioPortal [47,48,49]. From the CCLE, we analyzed gene-level copy number alterations (generated using the Genome Analysis Toolkit, GATK pipeline, hg38-aligned) and RNA sequencing expression data (quantified using the GTEx pipeline, reported as log2 (TPM + 1)). From TCGA, we utilized GISTIC2-based copy number calls and RNA Seq V2 RSEM expression data (log2-transformed and z-score normalized). Homozygous deletions were defined as copy numbers ≤ −1.5 in the CCLE and a GISTIC score of −2 in TCGA dataset, while low expression was defined as values below the 25th percentile in the CCLE and z-score < −1.5 in TCGA data.

**Figure 2:**
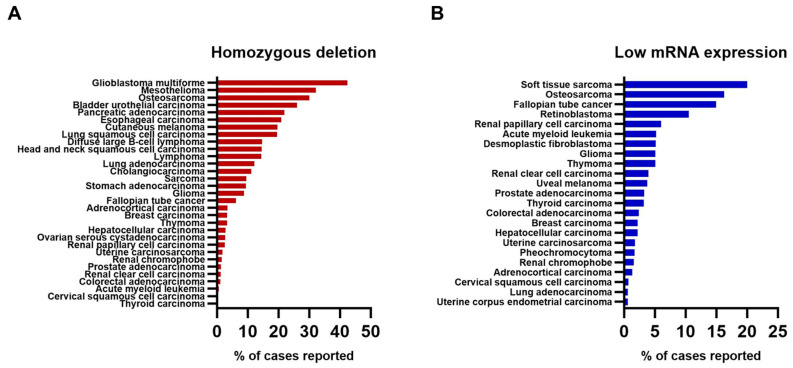
Incidence of MTAP loss in various cancer types. (**A**) Frequency of *MTAP* homozygous deletions and (**B**) low *MTAP* mRNA expressions obtained from the Cancer Cell Line Encyclopedia (CCLE DepMap Public 24Q4) and The Cancer Genome Atlas (TCGA) through cBioPortal.

Figure 2A shows the incidence of *MTAP* homozygous deletion in different cancer types based on the cases that have been reported in cBioPortal and the CCLE. The percentage of cases reported with homozygous deletion is highest in GBM (42%), followed by mesothelioma (32%) and osteosarcoma (30%). The rate of homozygous deletion is lowest in acute myeloid leukemia (0.5%), colorectal adenocarcinoma (0.3%), and prostate adenocarcinoma (0.2%). As for low mRNA expression, soft tissue sarcoma (20%), osteosarcoma (16%), and fallopian tube cancer (14%) showed the highest percentage of *MTAP* loss (Figure 2B). On the contrary, cervical squamous cell carcinoma (0.6%), lung adenocarcinoma (0.5%), and uterine corpus endometrial carcinoma (0.5%) have little loss of *MTAP* reported. These findings inform the importance of using *MTAP* as a biomarker for targeted therapy in different cancers. By identifying the frequency of *MTAP* homozygous deletion and low mRNA expression, clinical trials can be designed for specific cancers that show prominence in MTAP loss [50,51]. Currently, clinical trials of therapies targeted at tumors with MTAP loss are centered on solid tumors including sarcomas, carcinomas, and lymphomas with homozygous *MTAP* deletion [51,52,53]. In addition, GBM and urothelial cancers are some other forms of tumors that have been targeted [53,54,55].

### 2.4. Effects of MTAP Loss in Cancer

Loss of MTAP is intricately associated with multiple biological pathways including metabolism, the tumor microenvironment, and tumor progression. While researchers have seen contradicting perspectives on the effects of MTAP loss on tumorigenesis, most of these studies support the notion that MTAP deficiency contributes to tumor growth and proliferation through different mechanisms (Table 1).

MTAP is a known regulator of metabolic activities in cells, particularly in methionine salvage [18,71]. In GBM, loss of MTAP leads to altered methionine metabolism and increased consumption of methionine [72]. Furthermore, MTAP-deficient tumors exhibit intracellular and extracellular MTA accumulation in many different tumor types in vitro [7,18,56,57]. Beyond this general metabolic alteration, MTAP deficiency leads to enhanced expression of hypoxia-inducible factor 1α (HIF1-α) and activation of RIO kinase 1 (RIOK1) in pancreatic cancers, prompting metabolic adaptation towards a glycolytic phenotype and de novo purine synthesis [58]. Furthermore, MTAP loss triggers activation of ornithine decarboxylase (ODC), the enzyme that catalyzes the conversion of ornithine to putrescine and subsequently spermidine and spermine [60]. Elevation of polyamine levels results in enhanced tumorigenesis in *Saccharomyces cerevisiae*, pancreatic adenocarcinoma, neuroendocrine tumors, and breast cancer [59,60,61].

Besides metabolism, the role of MTAP in the immune microenvironment was observed in GBM tumors, where its deficiency prompted enhanced infiltration of M2 macrophages independent of interleukin-4 and interleukin-3 (IL4/IL3) signaling and mediated by the adenosine A_2B_ [62]. This subsequently created an immunosuppressive environment to promote tumor growth and survival [62]. Similarly, MTAP loss was found to upregulate immune checkpoint protein programmed cell death ligand 1 (PD-L1) in lung cancers, for subsequent inhibition of T-cell activity [63]. Reduced immune infiltrates with higher proportions of immunosuppressive cells and lower proportions of T lymphocytes and natural killer cells were observed in MTAP-null tumors [63].

MTAP also plays a role in regulating the cell lineage and morphology in different cancers. Loss of MTAP has been associated with enhanced cancer cell stemness in GBM as demonstrated by increased expression of prominin-1 (PROM1), also known as CD133, further elevating the tumorigenicity of these cells [10]. In GI cancers, *MTAP*-deleted tumor sections demonstrate epithelioid histology with higher mitotic rate [43].

Several studies highlight the impact of MTAP loss on tumor growth and aggressiveness. In HCC, MTA expression is associated with matrix metalloproteinase (MMP) and interleukin-8 (IL-8) transcription in HCC cells in vitro, accompanied by enhanced proliferation and activation of the transcription factor nuclear factor kappa-light-chain-enhancer of activated B cells (NF-κB) [56]. In GI cancers, MTAP-null tumors displayed a larger tumor size, higher proliferative index, and were associated with increased risk under the National Institutes of Health (NIH) consensus [43]. Furthermore, genes involved in hyperplasia such as fibroblast growth factor 3 (FGFR3) and phosphatidylinositol-4,5-bisphosphate 3-kinase (PIK3CA) were frequently mutated in MTAP-deficient bladder tumors, leading to poorer prognosis [12]. In T-cell leukemia, MTAP loss is associated with increased tumor aggressiveness and greater risk of tumor transformation to more malignant states [16]. Besides that, genomic structural variants affecting the *MTAP*-*CDKN2A* region in osteosarcoma were linked to amplified mouse double minute 2 (MDM2) expression, leading to inactivation of tumor protein p53 (TP53) and poor overall survival [64,65,66]. Analysis of TCGA GBM patient data also showed that progression-free survival (PFS) is significantly decreased in patients with *MTAP* deletion compared to patients with intact *MTAP* [10].

Accumulating evidence has demonstrated the role of MTAP in regulating tumor migration and invasion through different mechanisms. MTA-induced protein methyltransferase (PRMT) inhibition and lack of symmetric dimethyl arginine (SDMA) activity in MTAP-null lung cancers leads to reduced degradation of vimentin and increased metastatic activity [67]. Furthermore, knockdown of *MTAP* leads to activation of the glycogen synthase kinase 3 beta (GSK3β)/Slug/E-cadherin axis to promote migration and invasion of esophageal cancer cells [68]. Additionally, MTA accumulation and PRMT inhibition through loss of MTAP function promoted extracellular signal-regulated kinase (ERK)-mediated tumor metastasis in melanomas [69]. In MTAP-null breast cancers, an increased amount of putrescine led to enhanced metastatic activity [61].

While the above findings support the role of *MTAP* as a tumor suppressor gene, some studies challenge this notion. For example, loss of *MTAP* is associated with lower microsatellite instability and tumor mutation burden in colorectal cancer [46]. This is supported by prior studies in colorectal cancer that found a positive correlation between MTAP expression and tumor proliferation, migration, and invasion through epithelial–mesenchymal transition (EMT) [70]. In GBM cells, *MTAP* knockout did not increase cell proliferation, migration, and invasion as observed in other cancers [11]. Moreover, clinical data suggest that lower MTAP expression correlated with improved prognosis in adult GBM [11]. These findings underscore the complexity of the role of MTAP in cancer, suggesting that its impact may vary significantly depending on the tumor type and molecular context.

## 3. Therapeutic Targets and Strategies

**Figure 3:**
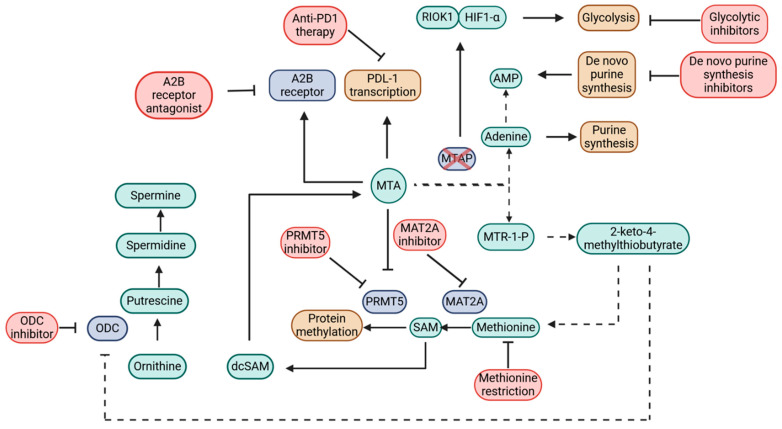
Schematic figure of synthetic vulnerabilities induced by MTAP loss and multiple therapeutic targets investigated over the years. Created in BioRender. Bavani Subramaniam. (2025) https://app.biorender.com/illustrations/67af92005139972ce02482a6. Abbreviations: AMP, adenosine monophosphate; dcSAM, decarboxylated S-adenosylmethionine; HIF1-α, hypoxia-inducible factor 1α; MAT2A, methionine adenosyltransferase 2A; MTA, methylthioadenosine; MTR-1-P, 5-methylthioribose-1-phosphate; ODC, ornithine decarboxylase; PD1, programmed cell death protein 1; PD-L1, programmed cell death ligand 1; PRMT5, protein methyltransferase 5; RIOK1, RIO kinase 1; SAM, S-adenosylmethionine.

Over the years, selective inhibition of *CDKN2A*/*MTAP*-deleted tumors has remained a significant challenge due to the absence of clinically effective therapeutic targets. Prior to the emergence of functional genomic tools, therapies for *MTAP*-deleted tumors primarily focused on exploiting vulnerabilities within the methionine metabolism pathways [7]. However, with advancements in RNA and Clustered Regularly Interspaced Short Palindromic Repeats (CRISPR)-based technologies, large-scale drug target discovery initiatives such as Project DRIVE and Project Achilles have revolutionized the field [7]. These projects concurrently identified protein methyltransferase 5 (PRMT5) as a key synthetic lethal vulnerability in *MTAP*-deleted cells, offering a promising avenue for targeted therapeutic development [71,73]. In this section, we will explore the different therapeutic targets identified over time (Figure 3), beginning with the recently discovered PRMT5/methionine adenosyltransferase 2A (MAT2A) axis and then tracing back to earlier targets that have shaped our understanding of *MTAP*-deleted tumor vulnerabilities.

### 3.1. Targeting Protein Methyltransferase 5 (PRMT5)

PRMT5 dependencies in MTAP-null tumors are one of the most studied therapeutic targets in recent years [18,71,73]. PRMT5 is a type II protein methyltransferase that catalyzes arginine methylation in the arginine–glycine or arginine–glycine–glycine (RG/RGG) motif [74,75]. SDMA catalyzed by PRMT5 is then recognized by Tudor domains in proteins, facilitating protein–protein interactions [76]. Loss of MTAP leads to accumulation of MTA, which competitively binds to the active domain of the PRMT5 enzyme, thereby increasing cancer cell dependence on PRMT5 [18,71,73].

#### 3.1.1. First-Generation PRMT5 Inhibitors

First-generation PRMT5 inhibitors were centered on S-adenosylmethionine (SAM)-competitive or non-SAM substrate-competitive inhibitors (Table 2) [77] and these were catered for patients with tumors regardless of MTAP status [78,79,80,81,82]. SAM-competitive inhibitors include JNJ-6469178, PF-06939999, PRT811, GSK3326595, PRT543, and LLY-283 [78,80,83,84,85,86]. These inhibitors bind to the SAM binding pocket found in the Rossman fold domain of PRMT5 to prevent methylation of downstream proteins [78,87]. JNJ-64619178 and PF-0693999 have been found to reduce cell proliferation and SDMA expression and modify alternative splicing of pre-mRNAs in pancreatic, hematological, breast, colon, lung, and ovarian cancers [78,80]. While the phase I clinical trial on JNJ-64619178 (NCT03573310) [88] is active, the phase I clinical trial on PF-0693999 (NCT03854227) [89] has been terminated by Pfizer due to strategic reasons not related to the safety profile or clinical responses [81]. PRT811, GSK3326595, PRT543, and LLY-283 have shown anti-metastatic and anti-tumor activity in preclinical models of brain tumors, colorectal carcinoma, non-small cell lung cancer (NSCLC), and melanoma [86,90,91,92]. Phase I clinical trials for PRT811 (NCT04089449) [53,84], GSK3326595 (NCT02783300) [52,85], and PRT543 (NCT03886831) [83,93] have been completed with a tolerable safety profile and some clinical activity.

Non-SAM-competitive inhibitors include T1551 and EPZ015666 that bind to PRMT5 active sites that are not SAM binding sites [82]. In preclinical studies, T1551 was found to reduce cell proliferation, downregulate oncogenes, and alter the phosphatidylinositol-3 kinase (PI3K)/serine/threonine kinase (AKT)/mammalian target of rapamycin (mTOR)/ERK signaling in NSCLCs [82]. EPZ015666 (GSK3235025) inhibited retinoblastoma cell proliferation and induced cell cycle arrest at the G1 phase [94]. These two drugs, however, have not been tested in clinical trials. While interest in PRMT5 inhibitors increased with the discovery of PRMT5 as a synthetic lethality in MTAP-null tumors, first-generation PRMT5 inhibitors were incapable of leveraging the MTA-rich environment of *MTAP*-deleted tumors, thus showing no specific inhibitory effects on *MTAP*-deleted tumors [7,71]. These drugs showed no robust selectivity for MTAP-null cancers [18,71,73], making second-generation inhibitors more suitable for targeting MTAP.

#### 3.1.2. Second-Generation Protein Methyltransferase 5 Inhibitors

Several studies have suggested that synergistic activity in MTA-accumulated cells will only occur if the PRMT5 inhibitor targets a PRMT5-MTA complex by binding to a separate site from the MTA binding site with zero interaction potency [7,107]. Hence, there has been a surge in MTA-cooperative PRMT5 inhibitors that have shown enhanced selectivity towards *MTAP*-deleted tumors (Table 2). Some of these, such as MRTX1719, TNG908, TNG462, AZD3470, and AMG193, are being extensively studied in clinical trials for multiple types of solid tumors [108,109,110,111,112]. These have shown marked anti-tumor activity across a range of solid tumors in preclinical studies including lung carcinoma, colorectal cancer, mesothelioma, bladder cancer, and GBM [95,97,98,99,112].

Clinical trials for MRTX1719 (NCT05245500) [108], TNG462 (NCT05732831) [110], AZD3470 (NCT06130553) [111,113], and AMG193 (NCT06333951,NCT06593522) [114,115] are still recruiting for patients with solid tumors, thoracic tumors, and NSCLC bearing *MTAP* deletion. The TNG908 clinical trial, while active, has ceased recruitment because initial studies show no partial response in all GBM patients tested [116]. While this molecule was designed to penetrate the blood–brain barrier, the lack of response in CNS tumors led to the design of follow-up candidate TNG456, currently undergoing preclinical evaluation [116].

Although MTA-cooperative PRMT5 inhibitors have shown extensive potential in multiple MTAP-null preclinical models, questions have been raised about the presumed accumulation of MTA in the presence of the tumor microenvironment. Barekatain and colleagues demonstrated that *MTAP*-deleted primary GBM tumors do not show significant accumulation of MTA in vivo, contrary to what was observed in vitro [117]. This discrepancy was attributed to the presence of an MTAP-expressing stroma that surrounds the *MTAP*-deleted tumors, further metabolizing MTA secreted by these tumors [117]. While this is the only published study that has shown a lack of MTA accumulation in human tumor tissues, it should be taken into consideration as a possible limitation for targeted therapy involving MTA-cooperative PRMT5 inhibitors.

Nevertheless, given that PRMT5 inhibitors are the prominent choice for the treatment of MTAP-null tumors [7,71,118], alternative strategies to utilize these inhibitors should be considered. Several studies have demonstrated the efficacy of PRMT5 inhibitors regardless of *MTAP* status, and these have proven beneficial in preclinical studies [119]. While these drugs do not utilize the MTA-rich environment, the efficacy of these drugs can be enhanced if basal PRMT5 activity in individual tumors can be determined through SDMA expression. Given that PRMT5 inhibitors were utilized to target tumors with low PRMT5 activity, they would be ideal candidates for clinical trials investigating the efficacy of these drugs [117]. The catch, however, still lies in identifying suitable biomarkers that can accurately depict low PRTM5 activity, a feat still unachievable in clinical settings.

Alternatively, the response towards PRMT5 inhibitors can be better gauged by assessing the metabolic profiles of individual patients. This is due to a difference in metabolic profiles of in vitro and in vivo models, whereby in the in vitro system, the culture media and nutrient availability, especially that of methionine and cysteine, can be tightly controlled to ensure MTA accumulation in MTAP-null tumors [120]. Such a case is not possible in patients, where nutrient depletion or surplus can drastically alter MTA levels, thus overestimating the efficacy of PRMT5 inhibitors [120]. A thorough understanding of metabolic profiles in patients with MTAP-null tumors would provide comprehensive insights into patient-to-patient variability in response towards PRMT5 inhibitors. This would also add the benefit of identifying novel combinations that target PRMT5 and specific metabolic pathways in these patients [121].

### 3.2. Targeting MAT2A

MAT2A dependency is the second most studied vulnerability in MTAP-null gliomas [7,18]. MAT2A catalyzes the conversion of methionine and ATP into SAM [122], which is the substrate required for PRMT5 activity [7,101]. When MAT2A is inhibited, PRMT5 is indirectly inhibited, and as such, it creates an equally good opportunity for targeting MTAP-null tumors [7,18].

Historically, methionine analogs such as cycloleucine have shown effective inhibitory effects in vitro but their poor pharmacokinetic properties and low potency have limited their in vivo application [123,124]. Stilbene derivates have also been identified as MAT2A inhibitors but have raised concerns due to their redox reactivity flags and reduced specific inhibitory effects [125,126]. Over the years, efforts to formulate MAT2A inhibitors were fraught with challenges as the enzyme was composed of a large hydrophilic active site and a spacious and highly hydrophobic allosteric binding site [57,127]. However, a reasonably efficacious allosteric MAT2A inhibitor, PF-9366, was identified, which unfortunately triggered a negative feedback upregulation of MAT2A that ultimately mitigated its potency [127]. In recent years, fragment-based screening and structure-guided design facilitated the discovery of allosteric MAT2A inhibitors with significantly improved efficacy that have been pushed for clinical trials [101,103]. These inhibitors are detailed in Table 2.

The earliest allosteric MAT2A inhibitors with reported efficacy were AG270 and IDE397, which have shown selective inhibition of MTAP-null colorectal cancer, pancreatic cancer, and other solid tumors [101,102]. Preclinical studies show anti-tumor activity of these agents and reduced SAM levels upon MAT2A inhibition [101,102]. Other MAT2A inhibitors include AZD9657, SCR-7952, BT115386, and FIDAS-5 [103,104,105,106]. These have also exhibited in vitro and in vivo anti-proliferative activities, p53 mediated apoptotic activity, reduced tumor stemness and lipogenesis, and inhibition of mTOR-mediated protein synthesis in *MTAP*-deleted tumors [103,104,105,106]. SCR-7952 also showed improved potency compared to AG270 with no elevation in bilirubin [104]. Clinical trials of AG-270 (NCT03435250) [51,128] on patients with advanced solid tumors or lymphoma with MTAP loss have been terminated due to liver toxicity and disease progression [129]. Clinical trials on S095035 (NCT06188702) [130], IDE397 (NCT04794699) [131,132], and ISM3412 (NCT06414460) [133] are currently active and recruiting for *MTAP*-deleted solid tumors. A clinical trial involving IDE397 in combination with AMG193 (NCT05975073) [134] is currently active for patients with MTAP-null solid tumors but not recruiting.

### 3.3. Targeting De Novo Purine Synthesis Pathway

The MTAP enzyme catalyzes the synthesis of adenine from MTA [13,71]. In the absence of MTAP, adenine nucleotides are synthesized by de novo biosynthesis through folate-mediated single-carbon metabolism [12,58]. As such, *MTAP*-deleted tumors have shown increased sensitivity to several inhibitors of de novo purine synthesis using antifolate agents and purine analogs (Table 3).

#### 3.3.1. Antifolate Agents

Pemetrexed is an antifolate agent that functions by suppressing dihydrofolate reductase, thymidylate synthase, and glycinamide ribonucleotide, which are key enzymes essential for folate metabolism [145]. In MTAP-deficient urothelial cancer cells, pemetrexed was found to induce DNA double-strand breaks, distort nucleotide pools, and trigger apoptosis at a significantly higher rate than in MTAP-proficient cells [12]. In vivo, pemetrexed was found to have anti-tumor effects on MTAP-deficient xenograft models [12]. Clinically, 43% of MTAP-deficient urothelial cancer patients showed response towards pemetrexed, although the trial was eventually closed due to changing efficacy of treatments (NCT02693717) [54]. However, since pemetrexed has been shown to increase immune cells and PD-L1 expression in MTAP-deficient tumors, it was hypothesized that it will synergize with immune checkpoint inhibitors (ICIs) for enhanced response [146]. Hence, pemetrexed in combination with avelumab, zimberelimab, and the A2A/A2B receptor antagonist etrumadenant are currently being studied in phase II clinical trials for MTAP-deficient urothelial cancers (NCT03744793/NCT05335941) [50,146,147,148].

Pralatrexate is another antifolate agent that inhibits dihydrofolate reductase and de novo purine synthesis via depletion of 10-formyl tetrahydrofolate [149,150]. Although there have been no reports of single pralatrexate use for MTAP-null tumors, it has been shown to be effective on MTAP-deficient T-cell acute lymphoblastic leukemia (T-cell ALL) when used in combination with 6-thioguanine [142]. However, the complete response in mice models was accompanied by pralatrexate-induced toxicity which was abrogated with the use of leucovorin (LV) rescue [142]. LV reduces the toxicity of pralatrexate by either competing for reduced folate carrier type 1 (RFC1) transport into cells, competing for polyglutamylation, or providing an alternate source of tetrahydrofolate [138].

#### 3.3.2. Purine Analogs

L-alanosine (ALA) is a purine analog and an antimetabolite that functions by inhibiting adenylosuccinate synthetase (ADSS), an enzyme involved in adenosine synthesis [151,152]. ALA was found to attenuate stemness in MTAP-deficient GBM cells by compromising mitochondrial function even at a low dose [135]. In addition, ALA sensitized GBM tumors to temozolomide in vitro and in vivo [135]. The sensitivity of ALA on MTAP-deficient tumors was also apparent in adult T-cell leukemia (ALT) [136]. Despite showing specific inhibitory effects on MTAP-null tumors, ALA failed in phase II clinical trials (NCT00062283) [153], for inducing hematologic toxicities in patients of different cancer types [152]. However, it has been suggested that a less rigorous regimen of ALA including a lower dose of treatment for a shorter duration will be beneficial in combination with a low dose of standard-of-care chemotherapy [135].

2-Fluoroadenine (2-FA) is another purine analog that shows specific inhibitory effects on MTAP-null tumors [13]. In *MTAP*-WT cells, MTA is converted to adenine, which further blocks the conversion of 2-FA into its toxic nucleotide by phosphorylation with 5-phosphoribosyl-1-pyrophosphate (PRPP) [13,137]. On the other hand, lack of adenine leads to a high level of PRPP and conversion of 2-FA into its toxic nucleotide in MTAP-null tumors, thus killing the tumor cells [13,137]. However, a limitation with this technique is that the drug targets normal dividing cells and cancer cells, creating non-specific toxicity on the host [137]. To overcome this, some studies have tested MTA in combination with 2-FA to ensure MTAP is able to protect normal cells by converting MTA to adenine, while MTAP-null tumor cells fail to carry out a similar process [137,154].

Several other purine analogs have been studied on *MTAP*-deleted tumors, including 6-thioguanine that targets de novo purine synthesis and kills tumor cells by incorporating 6-thioguanine nucleotides into DNA [155,156,157]. As mentioned previously, 6-thioguanine has also been tested in combination with MTA to prevent non-specific toxicity in normal cells [156,158]. Purine starvation has also been explored in MTAP-null tumors to prevent resistance towards purine analogues triggered by exogenous purine nucleotides [10].

### 3.4. Methionine Restriction

Loss of MTAP leads to reduced salvage of methionine and adenine from MTA in cancer cells, resulting in increased dependency on an exogenous supply of these nutrients (Table 3) [139]. To test this hypothesis, Batova and colleagues investigated the effects of methionine deprivation on MTAP-deficient T-cell ALL cells by removing this nutrient from the culture media [139]. This led to a 50% decrease in cell viability of MTAP-null cells as early as after 48 h of starvation without significant effects on *MTAP*-WT cells [139]. To eliminate the presence of methionine supply from other sources, such as non-dialyzed fetal bovine serum (FBS), Aoki and coworkers have tested the use of recombinant methioninase (rMETase) [143]. In their study, MTAP-null and *MTAP*-knockout (*MTAP*-KO) osteosarcoma cell lines were found to be more sensitive to rMETase than *MTAP*-wild type (*MTAP*-WT) cell lines [143], suggesting increased methionine dependency in cells without MTAP.

However, there have been several indications that methionine dependency is not solely reliant on MTAP status, and several cells have shown vulnerability to methionine restriction independent of MTAP status [120]. Preclinical studies have shown that methionine restriction inhibits tumor growth on rodent colorectal cancer models [159] and invasiveness of prostate cancer in mice models [160]. Methioninase has also been shown to be effective in patient-derived orthotopic xenograft (PDOX) models of Erwing sarcoma and melanoma, when used as a single drug or in combination with chemotherapy [161,162]. Although not many studies have made the comparison between *MTAP*-null and *MTAP*-WT tumors, there is still enormous potential in using methionine restriction as a technique to inhibit MTAP-null tumor growth as the methionine salvage pathway is significantly impacted in these tumors.

Clinical studies of methionine deprivation are more complicated as they require careful consideration of age, sex, nutrient requirements, and patient acceptance of methionine-restricted diets [163]. Methioninase has been shown to effectively deplete serum methionine levels in metastatic breast cancer patients [164], yet dietary methionine restriction is still being studied in several clinical trials. Standard-of-care therapy accompanied by dietary methionine restriction on gastric cancer and metastatic colorectal cancer patients showed an improved overall response rate and partial tumor response [165,166]. Additionally, researchers have suggested providing homocysteine in the diet to enable normal cells but not tumor cells to optimally synthesize methionine from homocysteine [167,168]. To take it a step further, using methioninase supplemented with homocysteine may be a better option in overcoming non-acceptance in dietary restrictions among some patients.

### 3.5. Glycolysis Inhibition

The role of MTAP in glycolysis has not been extensively studied, yet one study conducted on pancreatic tumors revealed enrichment of genes involved in the glycolytic pathway in patients with *MTAP* homozygous deletion (Table 3) [58]. To elucidate the specific role of *MTAP* deletion in glycolysis, the researchers showed increased glycolytic phenotypes in *MTAP*-deleted pancreatic tumor cells triggered by the upregulation in HIF1-α expression [58]. HIF1-α upregulation is mediated by RIOK1 stabilization activity, which further alters glucose metabolism in these tumors [58]. Following this, inhibition of glycolysis using 2-deoxy-D-glucose (2-DG) significantly reduced tumor growth in *MTAP*-KO preclinical models without significant inhibition in *MTAP*-WT models [58]. In addition, 2-DG showed synergistic anti-tumor activity when used in combination with ALA in these tumors [58].

### 3.6. Ornithine Decarboxylase Inhibition

In a number of studies associated with breast and pancreatic cancers, MTAP was found to regulate the activity of ornithine decarboxylase (ODC), the rate-limiting enzyme in the biosynthesis of putrescine (Table 3) [59,60,61]. Low MTAP expression led to increased ODC activity and perturbations in polyamine metabolism, further affecting the growth and metastasis of tumor cells [169,170]. To specifically inhibit the growth of breast cancers with low MTAP expression, Zhang and colleagues demonstrated the effect of difluoromethylornithine (DFMO) in inhibiting ODC and further reducing tumor migration, invasion, and angiogenesis in *MTAP*-knockdown (*MTAP*-KD) breast cancer cells [61]. Similarly, ODC inhibition using DFMO in MTAP-null pancreatic tumors led to cell growth inhibition and apoptotic activity [60].

### 3.7. Immune Checkpoint Inhibition

Immune checkpoint inhibitors (ICIs) in the treatment of MTAP-null cancers have garnered interest in the research field due to the synergistic effect of PRMT5 inhibition with anti-programmed cell death protein (PD1) drugs (Table 3). Chen and colleagues demonstrated that the PRMT5 inhibitor MRTX1719 sensitizes MTAP-null tumors to cytotoxic T-cell mediated lysis [171]. Moreover, combined PRMT5 and PD-1 inhibition led to enhanced in vivo anti-tumor activity [171]. In another study involving mouse models of liver HCC tumors, PRMT5 inhibition promoted lymphocyte infiltration and induced major histocompatibility complex II (MHC II) expression in the tumor microenvironment [172]. By combining PRMT5 inhibitor with anti-PD1 therapy, significant tumor regression and CD4^+^ and CD8^+^ upregulation were demonstrated in vivo [172]. Although this study was not specific to MTAP-null HCC, it supports the immunomodulatory potential of PRMT5 inhibition [172]

Clinically, a combination of pemetrexed (antifolate) and avelumab (anti-PD-L1) is currently being evaluated in MTAP-deficient metastatic urothelial carcinoma (NCT03744793) [50,146]. The challenge with targeting immune checkpoints in MTAP-deficient tumors lies in the MTA-rich environment that activates the adenosine A_2_B receptor to stimulate an immunosuppressive (M2) state in macrophages [62]. This motivated an improvised and active clinical trial with pemetrexed, zimberelimab (anti-PD1), and etrumadenant (dual antagonist of A2a and A2b receptors) (NCT05335941) [147,148] on MTAP-deficient advanced or metastatic urothelial cancers. Moreover, enzymatic depletion of MTA has been shown to restore T-cell activity and synergize with the PD-1 blockade, leading to improved tumor growth inhibition in vivo [144].

### 3.8. Unmet Needs

Despite years of research on MTAP, clinical trials (Table 4), and the multiple synthetic lethality associated with it, no specific treatment has been approved after clinical testing. While MTA-cooperative PRMT5 inhibitors are predicted to be the closest winner in the race, issues with MTA metabolism in the surrounding tumor microenvironment questions the actual applicability of an MTA-rich environment in PRMT5 inhibition. One of the major problems with this concept is the lack of an accurate preclinical model that recapitulates endogenous tumors. Histological analysis has shown that xenograft models do not exhibit a similar stromal cell population as compared to primary GBM tumor tissues [117]. Reproducible in vivo models with more “patient-like” features are essential to assertively translate findings from preclinical models into clinical trials. Genetically engineered or syngeneic mice models with spontaneous tumors bearing *CDKN2A*/*MTAP* homozygous deletion would significantly improve treatment modalities compared to xenograft models. While this is a challenging feat due to mouse developmental constraints [173], employing Cre-LoxP recombination systems to induce *CDKN2A*/*MTAP* deletion on specific tissues at certain developmental stages is a needed effort to precisely evaluate the efficacy of different therapeutic strategies in in vivo models.

Another major criterion in translating preclinical evaluation to clinical evaluation involves the selection of biomarkers of response and tumor regression. While MTA quantification has been employed extensively in cell culture media and in tumor tissues post-resection [117], comprehensive longitudinal studies to monitor disease progression are limited. At the same time, the efficacy of immunotherapy in an immunosuppressive microenvironment is unknown when MTA levels cannot be gauged. Recent studies on postoperative delayed neurocognitive recovery (dNCR) revealed lower preoperative and postoperative serum MTA levels in those who developed dNCR compared to those who did not [174]. A simple liquid chromatography–mass spectrometry (LC-MS) system was used to monitor MTA levels and determine its role as a biomarker of recovery in these patients [174]. By employing similar methods to study MTA as a biomarker of response and recovery in MTAP-null tumors, disease progression can be monitored in an actual clinical setting. In addition, the feasibility of using MTA-cooperative PRMT5 inhibitors can be better assessed with clinical data on MTA accumulation.

In cases where an MTA-rich environment is absent, shifting towards ICIs could be considered, as MTA stimulates an immunosuppressive tumor microenvironment [62]. In such cases, combination therapies using non-MTA-cooperative PRMT5 inhibitors that synergize well with anti-PD1 therapy require further studies for clinical translation. Additionally, studies on combination therapies involving inhibitors targeting mechanisms other than PRMT5 and MAT2A could be conducted with anti-PD1 therapy. A rational combination of therapies targeting different signaling pathways, coupled with a comprehensive evaluation of the metabolic and immune factors influencing MTAP-null tumors, would enable a more precise and effective precision medicine approach for these patients.

On another note, few studies have attempted to characterize the concurrent loss of *CDKN2A*, *CDKN2B*, and *MTAP*. While *CDKN2A* or *CDKN2B* deletion does not guarantee *MTAP* deletion, *MTAP* loss without *CDKN2A* or *CDKN2B* is a rare occurrence [175]. In addition, *CDKN2B* loss is always accompanied by *CDKN2A* loss, reflecting a deeper chromosomal loss at 9p21.3 [176]. Tumors harboring *CDKN2A* and *MTAP* co-deletions demonstrate elevated MTA, leading to vulnerability towards PRMT5 inhibition [7,18]. In the case of an additional deletion involving *CDKN2B*, the complete loss of p15^INK4B and p16^INK4A functions are expected to drive CDK4/6-mediated cell cycle progression and glycolysis [177]. Although direct comparative studies between tumors with these distinct genotypes are limited, complete loss of *CDKN2A*/B and *MTAP* represents a larger deletion of chromosome 9p21.3, which has been associated with greater genomic instability and poorer prognosis [14,176,177]. Stratifying patients based on these molecular subtypes could therefore improve precision therapy design, as triple deletions may represent tumors with increased malignancy and reduced therapeutic sensitivity, necessitating rational combination strategies.

## 4. Conclusions

*MTAP* is a versatile biomarker for targeted therapies in cancers with 9p21 loss, given its prominent role in regulating the metabolic, immune, and proliferative states of multiple tumors. Clinical diagnosis of MTAP loss has incorporated the use of multiple platforms to validate homozygous deletion and loss of protein expression. However, a greater emphasis on methylation studies is warranted to account for cases involving promoter hypermethylation, ensuring a more comprehensive assessment of MTAP deficiency. Furthermore, elucidating the impact of MTAP loss in different cancers will pave the way for novel therapeutic strategies based on specific tumor types, moving beyond a one-size-fits-all approach. The diverse role of MTAP appears to be context-dependent, varying across cancer types and cellular environments, which underscores the need to design interventions that are unique to each tumor type. Specifically, converging the already discovered synthetic lethality with other emerging targets hold promise for designing enhanced precision therapies.

Moving forward, several key challenges and opportunities remain in our pursuit of efficient therapies for MTAP-null tumors. Improving preclinical models to better recapitulate patient tumors will be critical for predicting accurate clinical responses. In addition, refining biomarker assessments to stratify patients will be crucial for monitoring disease and therapeutic efficacy. Moreover, optimizing combination therapies by integrating PRMT5/MAT2A inhibitors with immune checkpoint and metabolic modulators may uncover synergistic effects and improve patient outcomes. At the same time, a deeper understanding of the pharmacological effects of these inhibitors in clinical settings is warranted. By integrating functional genomics, drug discovery, and precision medicine, there is immense potential for translating preclinical discoveries into meaningful clinical benefits, ultimately enhancing therapeutic outcomes and quality of life for patients with *CDKN2A*/B and *MTAP* loss.

## Figures and Tables

**Table 1:** Effects of MTAP loss in cancer.

Effects of MTAP Loss	Cancer Type	Mechanism	References
Metabolism	Multiple	MTA accumulation in vitro	[7,18,56,57]
	Pancreas	Enhanced expression of HIF1-α and activation of RIOK1, metabolic adaptation towards glycolysis and de novo purine synthesis	[58]
	Pancreas, neuroendocrine, breast	Activation of ODC, elevation of polyamine levels	[59,60,61]
Immune microenvironment	GBM	Enhanced infiltration of M2 macrophages mediated by adenosine A_2B_ receptor	[62]
	Lung	Upregulation of PD-L1, inhibition of T-cell activity, upregulation of immunosuppressive cells	[63]
Cell lineage and morphology	GBM	Enhanced cancer cell stemness, increased PROM1/CD133 expression	[10]
	Gastrointestinal cancer	Epithelioid histology, high mitotic rate	[43]
Tumor growth and proliferation	Hepatocellular carcinoma	MTA-induced MMP and IL-8 transcription, enhanced proliferation, activation of NF-κB	[56]
	Gastrointestinal cancer	Large tumor size, high proliferative index, increased risk	[43]
	Bladder cancer	Mutation of genes involved in hyperplasia, e.g., *FGFR3*, *PIK3CA*	[12]
	T-cell leukemia	Increased tumor aggressiveness, malignant tumor transformation	[16]
	Osteosarcoma	Amplified MDM2 expression, inactivation of TP53, poor overall survival	[64,65,66]
	GBM	Decreased PFS	[10]
Tumor migration and invasion	Lung	Reduced degradation of vimentin, increased metastasis	[67]
	Esophageal cancer	Activation of the GSK3β/Slug/E-cadherin axis	[68]
	Melanoma	ERK-mediated tumor metastasis	[69]
	Breast	Increased putrescine, enhanced metastasis	[61]
Better prognosis	Colorectal cancer	Lower microsatellite instability and tumor mutation burden, reduced tumor proliferation, migration, invasion through EMT	[46,70]
	GBM	Reduced tumor proliferation, migration, invasion	[11]

Abbreviations: EMT, epithelial–mesenchymal transition; ERK, extracellular signal-regulated kinase; FGFR3, fibroblast growth factor receptor 3; GBM, glioblastoma multiforme; GSK3β, glycogen synthase kinase 3 beta; HIF1-α, hypoxia-inducible factor 1-alpha; IL-8, interleukin-8; MDM2, mouse double minute 2 homolog; MMP, matrix metalloproteinase; MTA, methylthioadenosine; NF-κB, nuclear factor kappa-light-chain-enhancer of activated B cells; ODC, ornithine decarboxylase; PD-L1, programmed death ligand 1; PFS, progression-free survival; PIK3CA, phosphatidylinositol-4,5-bisphosphate 3-kinase catalytic subunit alpha; PROM1/CD133, prominin-1 (also known as CD133); RIOK1, RIO kinase 1; TP53, tumor protein p53.

**Table 2:** Preclinical studies involving inhibitors of PRMT5 and MAT2A on MTAP-deleted tumors.

Inhibitors	Drugs	Manufacturer	Tumors	Response	References
SAM-competitive PRMT5 inhibitors	JNJ-64619178	Johnson & Johnson	Pancreatic, hematological, breast, colon, lung, and ovarian cancer	Reduced cell proliferation, increased alternative splicing burden	[78]
	PF-06939999	Pfizer	NSCLC	Reduced proliferation of NSCLC cells, dose-dependent decrease in SDMA levels, changes in alternative splicing of numerous pre-mRNAs	[80]
	PRT811	Prelude therapeutics	Brain tumors	Anti-tumor activity in mice models	[90]
	GSK3326595	GlaxoSmithKline	Colorectal carcinoma	Inhibited distant metastasis of CRC cells	[91]
	PRT543	Prelude therapeutics	NSCLC	Anti-tumor activity of NSCLC in vitro and in vivo models	[92]
	LLY-283	Eli Lilly	Melanoma	Anti-tumor activity on mouse xenografts	[86]
Non-SAM-competitive PRMT5 inhibitors	T1551		NSCLC	Reduced proliferation of cells, downregulated oncogene (*FGFR3* and *eIF4E*), interrupted PI3K/AKT/mTOR and ERK signaling	[82]
	EPZ015666 (GSK3235025)	Epizyme Inc./GlaxoSmithKline	Retinoblastoma	Inhibited retinoblastoma cell proliferation and led to cell cycle arrest at the G1 phase	[94]
MTA-cooperative PRMT5 inhibitors	MRTX1719	Mirati Therapeutics	Solid tumors	Antitumor activity across a panel of xenograft models (lung carcinoma, colorectal, mesothelioma) at well-tolerated doses	[95]
	MRTX9768	Mirati Therapeutics	Colon cancer	Inhibited SDMA and cell proliferation of HCT116 *MTAP*-deleted cells	[96]
	TNG908	Tango Therapeutics	Solid tumors including GBM	Tumor regression in MTAP-null cholangiocarcinoma, NSCLC, bladder cancer, DLBCL, and GBMs	[97]
	TNG462	Tango Therapeutics	Solid tumors	Significant potency and selectivity towards *MTAP*-deleted cells, durable pharmacodynamics modulation, tumor regression in vivo	[98]
	AMG193	Amgen	Solid tumors	Selective antitumor activity in MTAP-null models	[99,100]
MAT2A	AG-270	Agios Pharmaceutical	Colorectal cancer and pancreatic cancer	Reduced SAM levels in cancer cells and selectively blocked proliferation of MTAP-null cells both in tissue culture and xenograft tumors	[101]
	IDE397	IDEAYA Biosciences	Solid tumors	Anti-tumor activity in *MTAP*-deleted CDX and PDX of NSCLC, pancreatic, bladder, head and neck, esophageal, and gastric cancer	[102]
	AZD9567	AstraZeneca	Lymphoma, lung, and pancreatic cancer	Antiproliferative effects on *MTAP*-KO cells in vitro and in vivo, excellent preclinical pharmacokinetic properties	[103]
	SCR-7952	Jiangsu Simcere Pharmaceutical	Colorectal cancer	Significant in vitro potency and selectivity compared to AG270, robust in vivo anti-tumor activity with no elevation in bilirubin	[104]
	BT115386	ScinnoHub Pharmaceutical	Nasopharyngeal carcinoma	Activation of p53 pathway, induction of BAX apoptotic protein, promoted differentiation, suppressed stemness, inhibited lipogenesis, disrupted EBV latency in the *MTAP*-deleted NPCs, in vivo anti-tumor efficacy	[105]
	FIDAS-5		Multiple myeloma	Reduced cell proliferation and survival by inhibiting mTOR-mediated protein synthesis, improved bortezomib-based treatment, reduced in vivo tumor growth	[106]

Abbreviations: AKT, serine–threonine kinase; BAX, Bcl-2-associated X protein; CDX, cell line-derived xenograft; CRC, colorectal cancer; DLBCL, diffuse large B-cell lymphoma; EBV, Epstein–Barr virus; eIF4E, eukaryotic translation initiation factor 4E; ERK, extracellular signal-regulated kinase; FGFR3, fibroblast growth factor receptor 3; GBM, glioblastoma multiforme; MAT2A, methionine adenosyltransferase 2A; MTA, methylthioadenosine; MTAP, methylthioadenosine phosphorylase; mTOR, mammalian target of rapamycin; NPCs, neural progenitor cells; NSCLC, non–small cell lung cancer; PDX, patient-derived xenograft; PI3K, phosphatidylinositol-3 kinase; PRMT5, protein arginine methyltransferase 5; SAM, S-adenosylmethionine; SDMA, symmetric dimethylarginine.

**Table 3:** Preclinical studies involving other inhibitors on *MTAP*-deleted tumors.

Mechanism	Drugs	Tumors	Response	References
De novo purine synthesis (purine analogs)	ALA	GBM	Diminished stemness and compromised mitochondrial function in vitro, tumor regression in vivo, sensitized tumors to temozolomide	[135]
		Adult T-cell leukemia	Improved sensitivity towards *MTAP*-negative cell lines compared to *MTAP*-positive cell lines	[136]
		Pancreatic cancer	Synergistic effects with 2-DG on MTAP-null cells	[58]
	2-FA	GBM, lung	Combination of 2-FA and MTA showed robust tumor growth inhibition in vivo	[137]
	6-thioguanine	T-cell ALL	Anti-proliferative effects in vitro and in vivo	[138]
	Blocking nucleotide transporters or purine starvation	GBM	Low-toxicity purine synthesis inhibitor leads to extended survival and preferably depletes the CD133-positive subset of GBM cells	[10]
	ALA and MTA	T-cell ALL	Only growth of *MTAP*^−^ cells was inhibited, not *MTAP*^+^ cells	[139]
De novo purine synthesis (antifolate)	Pemetrexed	Urothelial carcinoma	Induced DNA double-strand breaks, distorted nucleotide pools, triggered apoptosis, anti-tumor effects on MTAP-deficient xenografts	[140]
		Lung	Clinically effective against *CDKN2A*/*MTAP*-null lung adenocarcinoma	[141]
	Pralatrexate and 6-thioguanine	T-cell ALL	Significant tumor regression in CEM xenografts [142]	[142]
Methionine restriction	rMETase	Osteosarcoma	*MTAP*-KO U2OS cells were more sensitive to rMETase than the parental *MTAP*-positive U2OS cells	[143]
	Removal of methionine from media	T-cell ALL	Reduced cell viability upon 48 h administration	[139]
Glycolysis	2-DG	Pancreatic cancer	Synergistic effects with ALA on MTAP-null cells	[58]
Ornithine decarboxylase	DFMO	Breast cancer	Inhibited ODC, reduced tumor migration, invasion, and angiogenesis	[61]
	DFMO	Pancreatic cancer	Tumor growth inhibition, apoptosis	[60]
Immune checkpoint	MTA-degrading enzyme	Melanoma	Increased TILs, impaired tumor growth, synergized with anti-PD1 therapy	[144]

Abbreviations: 2-DG, 2-deoxy-D-glucose; 2-FA, 2-fluoroadenine; ALA, L-alanosine; ALL, acute lymphoblastic leukemia; CD133, cluster of differentiation 133; *CDKN2A*, cyclin-dependent kinase inhibitor 2A; CEM, CCRF-CEM cell line; DFMO, difluoromethylornithine; GBM, glioblastoma multiforme; MTA, methylthioadenosine; MTAP, methylthioadenosine phosphorylase; ODC, ornithine decarboxylase; PD1, programmed cell death protein 1; rMETase, recombinant methioninase; TILs, tumor-infiltrating lymphocytes.

**Table 4:** Clinical trials targeted on patients with *MTAP*-deleted tumors.

Treatment	Target	Trial ID	Phase	Status	Date of Verification/Update	Patients	Actual/Estimated Enrollment	Primary Outcomes/Report	References
MRTX1719	PRMT5	NCT05245500	1	Active, recruiting	10 November 2025	Solid tumors with *MTAP* deletion	336 estimated	DLT, AE, ORR, DOR, PFS, OS, CSLA	[108]
TNG462	PRMT5	NCT05732831	1/2	Active, recruiting	6 May 2025	Solid tumors with *MTAP* deletion	225 estimated	Phase 1: MTD, DS Phase 2: Anti-neoplastic activity using RECIST v1.1 or mRECIST v1.1	[110]
TNG908	PRMT5	NCT05275478	1/2	Active, not recruiting	23 July 2025	Solid tumors with *MTAP* deletion	192 estimated	Phase 1: MTD Phase 2: Anti-neoplastic activity using RECIST v1.1 or mRECIST v1.1 or modified RANO criteria	[109]
AZD3470	PRMT5	NCT06130553	1/2	Active, recruiting	15 October 2025	MTAP deficient advanced/metastatic solid tumors	234 estimated	Phase 1: AE, SAEs, DLT	[111,113]
AMG193	PRMT5	NCT06333951	1	Active, recruiting	2 October 2025	Advanced thoracic tumors with homozygous *MTAP* deletion	500 estimated	Phase 1: DLT, TEAE, SAE	[114]
AMG193	PRMT5	NCT06593522	2	Active, recruiting	5 November 2025	Advanced non-small cell lung cancer	200 estimated	OR, TEAEs, EOIs, Cmax, Tmax, AUC	[115]
S095035	MAT2A	NCT06188702	1/2	Active, recruiting	16 October 2025	Advanced or metastatic solid tumors with deletion of *MTAP*	308 estimated	Phase 1: DLT, AE, SAE Phase 2: ORR	[130]
IDE397	MAT2A	NCT04794699	1	Active, recruiting	18 November 2025	Solid tumors harboring *MTAP* deletion	180 estimated	Phase 1: DLT, MTD, RP2D of IDE397 alone or in combination, ORR and DoR in combination expansion arms	[131,132]
ISM3412	MAT2A	NCT06414460	1	Active, recruiting	5 November 2025	Locally advanced/metastatic solid tumors with *MTAP* deletion	80 estimated	DLT, AE, RP2D	[133]
AMG193 and IDE397	PRMT5 and MAT2A	NCT05975073	1/2	Active, not recruiting	31 July 2025	Advanced MTAP-null solid tumors	53	Part 1: DLT, TEAE, SAE Part 2: OR, RECIST	[134]
AMG193 alone or in combination with docetaxel	PRMT5 and chemotherapy	NCT05094336	1/2	Active, recruiting	11 September 2025	Advanced MTAP-null solid tumors	649 estimated	Part 1 and 2: DLT, TEAE, AE, SAE Part 3: ORR	[26,27]
Pemetrexed and avelumab	Antifolate and immune checkpoint inhibitor	NCT03744793	2	Active, not recruiting	30 July 2025	MTAP-deficient metastatic urothelial cancer	18	ORR	[50,146]
Pemetrexed, zimberelimab, etrumadenant	Antifolate, immune checkpoint inhibitor, A2A and A2B receptor antagonists	NCT05335941	2	Active, recruiting	7 October 2025	Advanced or metastatic MTAP-deficient urothelial carcinoma	20 estimated	AE, CR, PR, BOR per RECIST v1.1	[147,148]
AG-270	MAT2A	NCT03435250	1	Terminated	25 July 2024	Advanced solid tumors or lymphoma with MTAP loss	123	Discontinued due to liver toxicity	[51,128,129]
ALA	De novo purine synthesis	NCT00075894	1/2	Completed	13 January 2009	MTAP-deficient high-grade recurrent malignant gliomas	18	N/A	[55]
ALA	De novo purine synthesis	NCT00062283	2	Completed	26 June 2013	MTAP-deficient cancers	65	No objective responses, grade 3/4 toxicities	[152,153]

**Abbreviations:** AE, adverse event; ALA, L-alanosine; AUC, area under the concentration-time curve; BOR, best overall response; Cmax, maximum concentration; CR, complete response; CSLA, clinically significant laboratory assessment; DLT, dose limiting toxicity; DoR, duration of response; DS, dosing schedule; EOIs, events of interest; MAT2A, methionine adenosyltransferase 2A; MTAP, methylthioadenosine phosphorylase; MTD, maximum tolerated dose; N/A, not available; OR, objective response; ORR, objective response rate; PFS, progression-free survival; PR, partial response; PRMT5, protein arginine methyltransferase 5; RECIST, Response Evaluation Criteria in Solid Tumors; RP2D, recommended phase 2 dose; SAE, serious adverse event; TEAE, treatment emergent adverse event; Tmax, time to maximum concentration.

## Abbreviations

The following abbreviations are used in this manuscript:

2-DG	2-Deoxy-D-glucose
2-FA	2-Fluoroadenine
ADSS	Adenylosuccinate synthetase
AKT	Serine/threonine kinase
ALA	L-alanosine
ALT	Adult T-cell leukemia
AMP	Adenosine monophosphate
ATP	Adenosine triphosphate
BAX	Bcl-2-associated X protein
CCLE	Cancer Cell Line Encyclopedia
CD133	Cluster of differentiation 133
CDK	Cyclin-dependent kinase
CDKN2A/B	Cyclin-dependent kinase inhibitor 2A/B
CDX	Cell-derived xenograft
CNA	Copy number alterations
CNS	Central nervous system
CNV	Copy-number variations
CRC	Colorectal carcinoma
CRISPR	Clustered regularly interspaced short palindromic repeats
CSF	Cerebrospinal fluid
ddPCR	Droplet digital polymerase chain reaction
DFMO	Difluoromethylornithine
DLBCL	Diffuse large B-cell lymphoma
dNCR	Delayed neurocognitive recovery
EBV	Epstein–Barr virus
eIF4E	Eukaryotic translation initiation factor 4E
EMT	Epithelial–mesenchymal transition
ERK	Extracellular signal-regulated kinase
FBS	Fetal bovine serum
FGFR3	Fibroblast growth factor 3
FISH	Fluorescence in situ hybridization
GBM	Glioblastoma
GI	Gastrointestinal
GSK3β	Glycogen synthase kinase 3 beta
HCC	Hepatocellular carcinoma
HIF1-α	Hypoxia-inducible factor 1α
ICI	Immune checkpoint inhibitor
IHC	Immunohistochemistry
IL	Interleukin
KD	Knockdown
KO	Knockout
LC-MS	Liquid chromatography–mass spectrometry
LV	Leucovorin
MAT2A	Methionine adenosyltransferase 2A
MDM2	Mouse double minute 2
MHC II	Major histocompatibility complex II
MLPA	Multiplex ligation-dependent probe amplification
MMP	Matrix metalloproteinase
MPM	Malignant pleural mesothelioma
MTA	Methylthioadenosine
MTAP	Methylthioadenosine phosphorylase
mTOR	Mammalian target of rapamycin
MTR-1-P	5-methylthioribose-1-phosphate
NF-κB	Nuclear factor kappa-light-chain-enhancer of activated B cells
NGS	Next-generation sequencing
NIH	National Institutes of Health
NPC	Neural progenitor cell
NSCLC	Non-small cell lung cancer
ODC	Ornithine decarboxylase
PCAWG	Pan-Cancer Analysis of Whole Genomes
PD-1	Programmed cell death protein 1
PD-L1	Programmed cell death ligand 1
PDOX	Patient-derived orthotopic xenograft
PDX	Patient-derived xenograft
PFS	Progression-free survival
PI3K	Phosphatidylinositol-3 kinase
PIK3CA	Phosphatidylinositol-4,5-bisphosphate 3-kinase
PRMT	Protein methyltransferase
PRMT5	Protein methyltransferase 5
PROM1	Prominin-1
PRPP	5-phosphoribosyl-1-pyrophosphate
RFC1	Reduced folate carrier type 1
RG	Arginine–glycine
RGG	Arginine–glycine–glycine
RIOK1	RIO kinase 1
rMETase	Recombinant methioninase
SAM	S-adenosylmethionine
SDMA	Symmetric dimethyl arginine
T-cell ALL	T-cell acute lymphoblastic leukemia
TCGA	The Cancer Genome Atlas
TIL	Tumor-infiltrating lymphocyte
TP53	Tumor protein 53
UC	Urothelial cancer
